# A quantitative study of 3D-scanning frequency and Δd of tracking points on the tooth surface

**DOI:** 10.1038/srep14350

**Published:** 2015-09-24

**Authors:** Hong Li, Peijun Lyu, Yuchun Sun, Yong Wang, Xiaoyue Liang

**Affiliations:** 1 Center of Digital Dentistry, Peking University School and Hospital of Stomatology, Faculty of Prosthodontics, Peking University School and Hospital of Stomatology, National Engineering Laboratory for Digital and Material Technology of Stomatology, Research Center of Engineering and Technology for Digital Dentistry, Ministry of Health, 22 Zhongguancun Nandajie, Haidian District, Beijing 100081, China; 2 Beihang University, School of Instrumentation Science and Opto-electronics Engineering, Xue Yuan Road no. 37, Haidian District, Beijing 100191, China

## Abstract

Micro-movement of human jaws in the resting state might influence the accuracy of direct three-dimensional (3D) measurement. Providing a reference for sampling frequency settings of intraoral scanning systems to overcome this influence is important. In this study, we measured micro-movement, or change in distance (∆d), as the change in position of a single tracking point from one sampling time point to another in five human subjects. ∆d of tracking points on incisors at 7 sampling frequencies was judged against the clinical accuracy requirement to select proper sampling frequency settings. The curve equation was then fit quantitatively between ∆d median and the sampling frequency to predict the trend of ∆d with increasing f. The difference of ∆d among the subjects and the difference between upper and lower incisor feature points of the same subject were analyzed by a non-parametric test (α = 0.05). Significant differences of incisor feature points were noted among different subjects and between upper and lower jaws of the same subject (P < 0.01). Overall, ∆d decreased with increasing frequency. When the frequency was 60 Hz, ∆d nearly reached the clinical accuracy requirement. Frequencies higher than 60 Hz did not significantly decrease Δd further.

Primary oral CAD/CAM data is generally composed of 3D point cloud dentition and soft tissue surface data. This 3D data can be acquired using either an indirect or a direct method. For the indirect method, a traditional impression is made clinically from which a plaster model is measured and designed. For the direct method, the subject’s dentition and soft tissue surface are measured directly inside the mouth, without the creation of an impression. Intraoral optical scanning technology can be utilized as part of the direct method to acquire 3D point-cloud dentition and soft tissue surface data without actually making contact with the subject. Moreover, this technology has two prominent characteristics: ① the subjects are human beings, and ② the subjects are not contacted with the scan wand.

There is, however, an important cause of error associated with intraoral scanning. When subjects are asked to remain motionless during the procedure, the human body will still always move slightly and continuously. This “micro-movement” consists of voluntary motion (e.g., respiration or swallowing) and involuntary motion (e.g., heartbeat, arterial pulse or other myoelectric physiological activities). Micro-movement of dentition and soft tissue during the intraoral scanning procedure will, naturally, produce errors in scanning measurement. Additionally, the absence of contact of the subject with the scan wand during this procedure further allows micro-movement to complicate measurement. In the indirect method, during traditional impression making, doctors stabilize the rigid tray using their fingers to ensure a tight contact between the impression material and the tissue surface, thus reducing the impact of micro-movement on impression accuracy. During the model scanning procedure, the plaster model is motionless, thus avoiding much of the associated error. That being said, the level of accuracy of each of the two methods is tends to be similar at ~20 μm^1^, though micro-movement in the direct method causes it to be slightly less accurate than that in the indirect method, according to multiple studies using different scanning systems^1^,^2^,^3^,^4^,^5^,^6^,^7^,^8^,^9^.

Acquisition of a 3D digital impression consists of the generation of a single 3D image and then the merging of multiple 3D images. Generation of the first single 3D image via intraoral scanning can be accomplished using either of two general methods^10^: ① obtaining 3D surface data using a point-to-point or layer-to-layer method at a very high frequency (>10,000 Hz) using confocal microscopy technology, active wavefront sampling and optical coherence tomography; and ② merging the single 3D image on the base of multiple 2D images using triangulation and moiré interferometry. Importantly, micro-movement affects both steps of this procedure. In method 1, micro-movement results in errors of relative position among points or layers and, in method 2, among relative 2D images.

The most direct way to overcome the influence of micro-movement is by increasing the sampling frequency, or the acquisition rate for creation of a single 3D image. However, issues persist with this workaround: ① An ambiguous definition of sampling frequency, mainly regarding a point, layer or 2D image, may not accurately reflect the ability of particular scanning instrument to overcome micro-movement; ② a higher sampling frequency requires a higher intensity of light, leading to an increased possibility of tissue damage; and ③ a higher sampling frequency requires better equipment performance, leading to higher costs and difficulties promoting the use of intraoral scanning systems. Therefore, establishing an appropriate frequency for intraoral scanning is necessary. However, studies related to this issue have not yet been reported in the stomatology field.

The skull and torso are connected by the spine, the mandible is connected to the maxilla through the temporomandibular joint and ligaments, and each of these groups has a particular degree of mobility and range of motion. Moreover, the mandible can hinge axis motion, slide or remain stationary relative to the maxilla. With such a complex design, the impact of micro-movement in different positions of the arch likely varies.

This study is a quantitative analysis between sampling frequency and micro-movement during the generation of a single 3D image in stationary subjects that seeks to provide a reference for setting the sampling frequency of an intraoral scanning system.

## Methods

This study was approved by the bioethics committee of Peking University School and Hospital of Stomatology (PKUSSIRB-201412008) and was carried out in accordance with approved guidelines for human subjects research. The procedures and risks involved with participation in this study were discussed with the volunteers, and written informed consent was obtained from each included participant.

### Materials

Arti-Spray^®^ Occlusion Articulating Spray (Patterson, Germany); Mouth prop: OptraGate (Ivoclar Vivadent, Sweden); Flowable Resin: Filtek Z350 XT (3M ESPE, America); 3D Printing Resin (e-shell300, EnvisionTEC, Germany); Mark Point Slice (Shenzhen Branch Innovation Times Electronic Co., Ltd., China).

### Instruments

Optical Tracking System (Beihang University Precision Opto-mechatronics Technology, Key Laboratory of Education Ministry, China) (CCD resolution: 1040 × 1088 pixels; Spatial resolution: 174.2 μm; Measurement precision: 16 μm^11^); Computer configuration: 8 cores clocked at 1.7 GHz, 16 G memory and discrete graphics; Dental optical rapid prototyping machine (EnvisionTEC, Germany); Intraoral scanner (Organical, R + K CAD/CAM Technologie GmbH & Co. KG, Germany).

### Methods

Five volunteers were included in this study, aged 27 to 30 years old, in good health, with no clear causes of involuntary systemic tremor symptoms, no alcohol habitat or physical habitual action, no severe respiratory symptoms, and normal temporomandibular joint after examination. The study was conducted in a dust-free environment with a suitable temperature (26 ~ 28 °C) and natural light time (10:00 ∼ 15:00; sunny).

**Figure 1:**
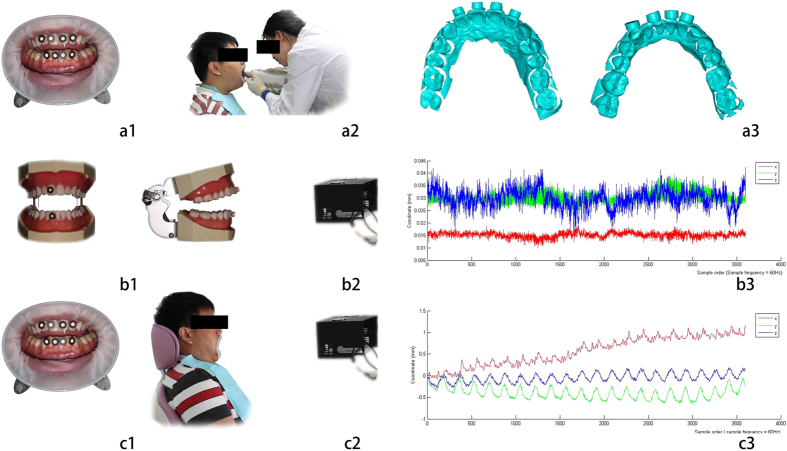
Data acquisition. (**a**) 3D data acquisition for building the rigid body: (**a1**) mark points distribution, (**a2**) 3D data acquisition, (**a3**) 3D data; (**b**) trajectory data acquisition of track points on motionless dental model: (**b1**) mark points distribution, (**b2**) data acquisition, (**b3**) trajectory data; (**c**) trajectory data acquisition of track points on volunteers: (**c1**) mark points distribution, (**c2**) data acquisition, (**c3**) trajectory data.

Dental resin cylinders (φ5 × 3 mm, 0.1 g) were manufactured using the dental optical rapid prototyping machine. A slice of paper was pasted on the end of each cylinder with its center acting as the cylinder’s center mark point for optical tracking. Subjects were given a mouth prop to distract the lips and expose the incisors, which were kept dry (Fig. 1). Four positioning cylinders were then affixed to the labial surfaces of four incisors per jaw with flowable resin. One cylinder was placed on a stationary dental model. Mark points on the stationary dental model were tracked as a group, and the resulting dataset was used as the control.

Subjects were asked to sit in a dental chair with their back and head on a rigid support with eyes closed, mouth opened naturally and normal breathing, and they were also asked to not to swallow saliva during the measurement (shop towels were worn). The procedure was repeated three times at three different sampling frequencies (60, 150 and 300 Hz) using the optical tracking system, and 3,600 groups of coordinates were obtained during each repeat. The same procedure was utilized for recording micro-movement tracks of the stationary dental model. All measurements were completed within 10 minutes, and all data were outputted as a TXT format dataset. The relative spatial descriptions of the x-axis, y-axis and z-axis of the machine coordinate system when facing the subject were left and right, up and down and front and back, respectively. Micro-movement tracking was re-sampled at 60 Hz at four intervals (11, 5, 3 and 2 track points) to obtain the track point trajectory at four sampling frequencies: 5, 10, 15 and 20 Hz. The 3D surface shape data of dentition and cylinder center points were obtained using the intraoral scanner.

**Figure 2:**
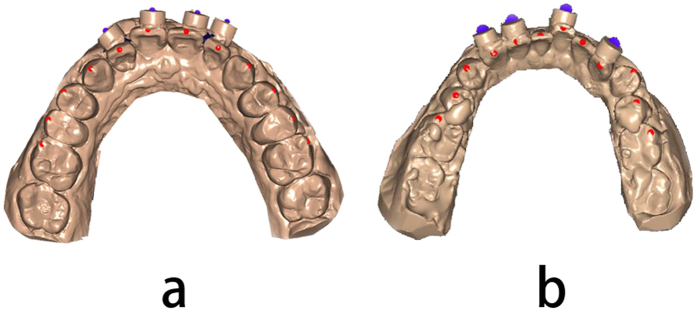
Track points fitting and feature points selection. (**a**) Upper jaw; (**b**) lower jaw.

The ‘feature’ points of each tooth between the first molars on a jaw were identified based on the 3D dentition data; these feature points included the incisor edge midpoints, canine teeth spire points, premolar buccal spire points and the first permanent molar spire points (Fig. 2). The partial coordinate system was established on the basis of four non-coplanar tracking points, and the geometric center of the four was defined as the origin point. The spatial coordinates of each feature point were calculated automatically, and the distance between each feature point (Δd) was obtained using MATLAB software according to coordinate transformation between the machine coordinate system and the partial coordinate system and vectors from the origin point to each feature point in the partial coordinate system.

First, Δd of the upper and lower right central incisor feature points scanned at the seven sampling frequencies mentioned above were measured for calculating the proportion (%) of each Δd that was less than 100, 60, 40, 30 and 20 μm at each sampling frequency. The quantitative equation was then created with median Δd(M) as the dependent variable and sampling frequency (f) as the independent variable. Secondly, the coordinates of a series of track points were separated using the operator-based null space pursuit (NSP) algorithm^12^. Proportions of Δd of each component were computed for analyzing their corresponding clinical effects. Lastly, the range of Δd of dentition on a jaw was obtained based on the Δd of all feature points at the 60 Hz sampling frequency. The results were put into SPSS 20.0. After analyzing the normality of Δd of each trajectory, non-parametric tests were used to evaluate the variance of the Δd from maxillary and mandibular jaws and among different subjects (α = 0.05). All signal analyses were handled in the same manner.

## Results

**Figure 3:**
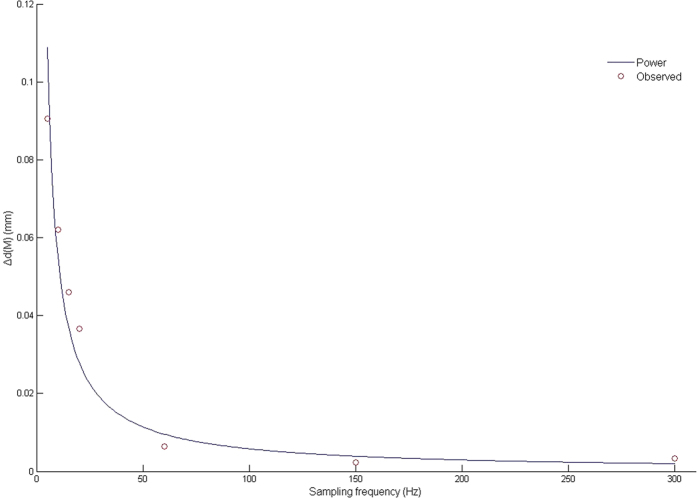
Curve estimation of regression analysis.

Every Δd value showed a right-tailed positive skew and non-normal distribution (one-sample Kolmogorov-Smirnov test, P < 0.05). Parameters of upper and lower right incisor feature micro-movement trajectory at seven sampling frequencies are shown in Table 1. Median Δd(M) and the sampling frequency (f) satisfy the power curve equation: ∆d(M) = 0.526 × f ^−0.979^ (f ∈ [5, 300]) (R^2^ = 0.937, P < 0.001) (Fig. 3).

**Figure 4:**
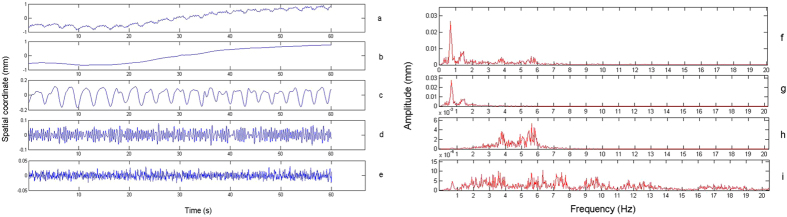
Trajectory decomposition. Time domain: (**a**) original data, OD; (**b**) trend term, TT; (**c**) the first principle component, PC-1; (**d**) the second principle component, PC-2; (**e**) residual component, RC; Frequency domain: (**f**) OD–TT; (**g**) PC-1; (**h**) PC-2; (**i**) RC.

**Figure 5:**
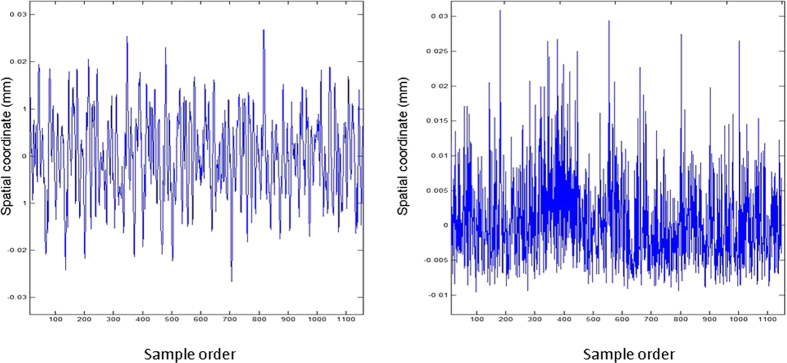
Stationary trajectory component (left) versus residual trajectory component (right).

**Figure 6:**
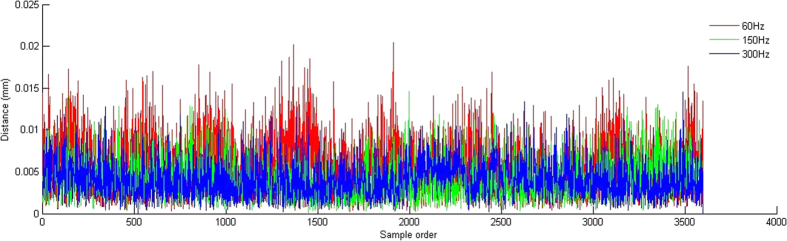
Δd of model trajectory at three sampling frequencies.

**Figure 7:**
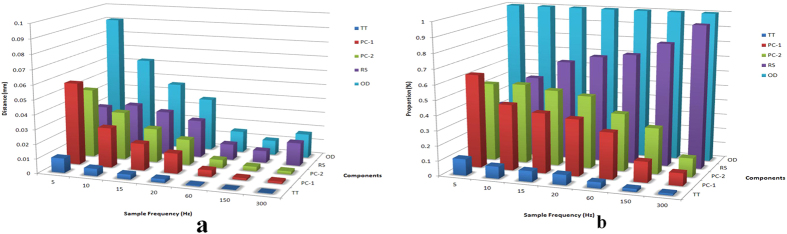
Change of (a) Δd value and (b) proportion of components with increasing sampling frequency.

Figure 4 shows that the trajectory can be divided into four components: ① the trend term (TT), which has low repeatability and refers to changes occurring over the course of the entire procedure relative to the base line; ② the first principal component (PC-1), which has a similar frequency to a breath at an average of 0.31 Hz; ③ the second principal component (PC-2), which has an average frequency of 2.21 Hz, or 2–4 times the heart rate; and ④ the residual component (RC), which has a main frequency range of 1 ~ 20 Hz, which is almost in the same range as the masticatory muscle α motor neuron fuse frequency, and an amplitude range of 10 μm (Fig. 5). Δd (mean ± SD) values of the stationary model at 60, 150 and 300 Hz were 6.1 ± 1, 4.9 ± 0.8 and 4.7 ± 0.6 μm, respectively, which were significantly different than those from the RC of volunteers (Wilcoxon (W) test, P < 0.05) (Fig. 6). Figure 7 shows that overall Δd decreased with increasing f when sampling frequency was less than 60 Hz. When sampling frequency was greater than 60 Hz, overall Δd did not change much. Specifically, RC increased gradually with increasing f past 60 Hz, while the three remaining components decreased gradually.

**Figure 8:**
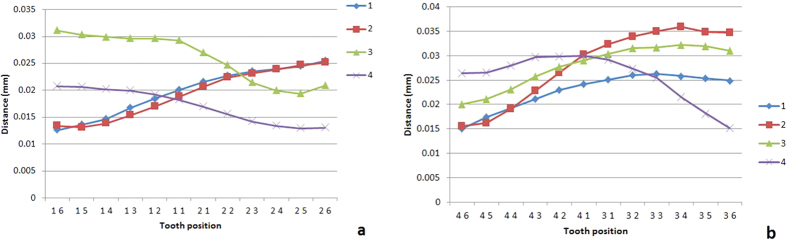
∆d value of different tooth positions (f = 60 Hz). (**a**) Upper jaw; (**b**) lower jaw.

The median Δd at different tooth positions changed gradually with a range of less than 30 μm and maxima located at the left end, right end or middle of the arch (Fig. 8). The range of Δd variation of the entire dentition can be estimated by Δd of the central incisor (±15 μm).

At the same sampling frequency, there was a significant difference (Kruskal-Wallis H (K) test, P < 0.01) in Δd among different volunteers. Δd between the upper and lower jaw of the same volunteer was also significantly different (Wilcoxon (W) test, P < 0.01).

## Discussion

The human trajectory of micro-movement includes both rhythmic and non-rhythmic components. The high-frequency rhythmic component is similar to a sinusoidal signal, whose frequency can be up to 20 Hz. According to the Nyquist-Shannon sampling theory (sampling frequency should be at least twice the frequency of the signal) and sinusoidal signal sampling requirements (sampling frequency should at least three times the frequency of the signal)^13^, the sampling frequency was set at a minimum of 60 Hz to fully reveal the characteristics of the trajectory. The range of sampling frequencies of intraoral scanning systems currently used in the clinic typically falls between 5 and 20 Hz. As each sampling process is undertaken independently, to acquire new tracks corresponding to this range of frequency at 60 Hz, a repeated sampling was done in this study.

The fact that every Δd of a micro-movement signal corresponding with its sampling rate was right-tailed positive skewed shows that human micro-movement speed is different at the same time intervals; thus high-velocity micro-movement would result in greater degree of measurement error than low-velocity micro-movement over the same time interval. Simply increasing sampling frequency is not the most efficient way to reduce the error caused by Δd. Scanning accuracy is the most important factor affecting the final effects and longevity of restoration, and it requires meeting two specific requirements: an acceptable range of crown margin whose maximum limit is 100 μm^14^ and physiological tooth mobility of 40 ~ 80 μm^15^. Given that the typical accuracy of a model scanner is 20 μm and understanding that Δd decreases as f increases, this study utilized five levels of evaluation (100, 60, 40, 30 and 20 μm) within the range of 20–100 μm to study the percentage of Δd meeting each level. Various errors of design and manufacturing are also involved in CAD/CAM, therefore the five standard Δd levels above were selected to reach the minimum standards of human micro-movement. To ensure the accuracy of the final restoration, further research on the exact requirements of Δd is needed.

Frequency components of human micro-movement trajectory, except for RC, were less than 5 Hz (the minimum standard), so ∆d of these three components all decreased with f increasing. ∆d of RC was stable when f was less than 20 Hz and decreased dramatically when f was greater than 20 Hz. The discharge frequency of a motor neuron in masticatory muscle α is generally between 5 and 25 Hz, which is the same as the frequency of RC. Thus, we speculate that the high-frequency, low-amplitude vibration evident by RC is generated by muscle electrical activity^16^. With an increase in f, overall Δd decreased; the proportion of the high-amplitude, low-frequency components of Δd decreased; and the proportion of the low-amplitude, high-frequency components of Δd increased.

Measurements of the subjects showed that overall Δd was lower at 150 Hz than at 300 Hz. One possible reason for this difference might be the increasing sensitivity of measuring instruments as the environmental light source changes. What’s more, there is no statistic difference between Δd at 150 Hz and 300 Hz of the five volunteers. Measurement at 150 Hz was followed by the measurement at 300Hz. Because there is only a short break between the two measurements process, muscle fatigue might be considered as another reason for the result. However, on the stationary model, Δd at 150 Hz and 300 Hz was similar and was lower than that at 60 Hz. The reasons for this difference should be further studied.

In this study, the conditions were that the subjects be at rest with the mouth open naturally and that the subjects were not to be affected by the doctor’s hand acting as a fulcrum (three fingers on the wand and one finger on the patient’s tooth or face for support). However, the subject likely contrasts the force transported by doctor’s fingers in or near the mouth during the actual process, thus potentially enlarging the velocity and amplitude of human micro-movement and leading to larger ∆d than the results in this study show. In future studies, we will improve the test situation to make it closer to the actual clinical process.

In this study, the relationships between upper and lower jaws for the median ∆d(M) and interquartile (Q) of micro-movement trajectory of five volunteers were shown to be M_UpperJaw_ > M_LowerJaw_ and Q_UpperJaw_ < Q_LowerJaw_, respectively, which demonstrates that physiological movement like breath and heartbeat have less of an impact on mandibular micro-movement as the temporomandibular joint and masticatory muscle can buffer the impact. Q was relatively larger for the lower jaw because the flexible structure of the temporomandibular joint leads its unstable positioning.

The range of Δd on a dentition was less than 30 μm, with maximum values seen at the ends or middle of an arch. The main factor affecting the distribution of Δd is suspected to be body movement, which presents as the non-rhythmic component (TT). During the actual scanning procedure, the range of Δd may be enlarged due to increased rotation and slide of the lower jaw caused by outer forces from the doctor, thus the likelihood of the maximum value occurring at the incisor area is increased.

In this study, Δd of ‘stationary’ human micro-movement was measured using an optical tracking system. In clinical applications, the doctor holds the scan wand to scan the 3D shape of dentition and other oral tissues. Hand shake is, therefore, likely another source of error which will be studied in further experiments.

## Conclusion

The clinical restoration accuracy of an intraoral scanning system can be met when its sampling frequency is 60 Hz. However, increasing sampling frequency beyond 60 Hz does not significantly decrease the Δd of micro-movement further.

## Additional Information

**How to cite this article**: Li, H. *et al.* A quantitative study of 3D-scanning frequency and ∆d of tracking points on the tooth surface. *Sci. Rep.* **5**, 14350; doi: 10.1038/srep14350 (2015).

## Figures and Tables

**Table 1:** Parameters of ∆d in five volunteers per sampling frequency.

**Jaw**	**F (Hz)**	**M (μm)**	**Q (μm)**	**Min (μm)**	**Max (μm)**	**R (μm)**	**95% CI (μm)**	**Proportion (%)**				
								**∆d < 100 μm**	**∆d < 6 0μm**	**∆d < 4 0μm**	**∆d < 3 0μm**	**∆d < 20 μm**
Upper Jaw	5	85.7	64.4	4.4	711.1	706.7	91.9–106.1	59.9	27.4	10	5.7	2.3
	10	60.1	45.4	6.6	270.3	263.7	56.1–64.4	85.0	49.6	24.6	13.0	4.3
	15	47.8	31.9	6.1	275.1	269.0	45.6–50.5	95.8	70.4	38.0	21.3	7.3
	20	37.6	26.1	3.1	134.1	131.0	34.4–40.0	98.8	85.9	55.1	34.1	14.0
	60	14.4	9.2	1.6	41.5	39.9	14.1–14.9	100	100	99.8	95.9	77.1
	150	15.5	10.8	1.0	49.3	48.3	15.3–15.9	100	100	99.6	93.9	67.8
	300	16.5	11.5	1.7	45.0	43.3	16.1–16.8	100	100	98.8	91.6	64.9
Lower Jaw	5	90.5	70.4	2.9	530.6	517.7	84.1–97.5	54.8	25.9	12.2	7.0	2.2
	10	62.1	44.8	3.2	245.2	242.0	57.9–65.7	84.6	49.6	24.3	13.4	5.4
	15	46.0	32.0	4.7	154.0	149.3	43.1–48.9	95.0	70.8	40.4	22.4	7.3
	20	36.6	24.3	1.7	141.7	140.0	34.4–38.6	98.2	84.8	54.8	34.7	14.4
	60	6.4	10.2	0.1	65.6	65.5	6.2–6.6	100	99.9	98.8	94.4	74.0
	150	2.3	6.0	0.1	42.9	42.8	2.2–2.4	100	100	99.9	98.7	90.6
	300	3.3	13.5	0.1	58.9	58.8	3.1–3.5	100	100	97.2	87.5	62.1

Median Δd (M), interquartile range (Q), maximum (Max), minimum (Min), range (R), median 95% confidence interval (95% CI). At each frequency, the proportion (%) of 3,599 measured Δd values that were less than each of the five standards (100, 60, 40, 30 and 20 μm) is shown.

## References

[b1] Ender A. & Mehl A. Accuracy of complete-arch dental impressions: A new method of measuring trueness and precision. J. Prosthet. Dent. 109, 121–128 (2013). 23395338 10.1016/S0022-3913(13)60028-1

[b2] Patzelt S. B., Emmanouilidi A., Stampf S., Strub J. R. & Att W. Accuracy of full-arch scans using intraoral scanners. Clin. Oral Investig. 18, 1687–1694 (2014). 10.1007/s00784-013-1132-y 24240949

[b3] Andriessen F. S., Rijkens D. R., van der Meer W. J. & Wismeijer D. W. Applicability and accuracy of an intraoral scanner for scanning multiple implants in edentulous mandibles: A pilot study. J. Prosthet. Dent. 111, 186–194 (2014). 24210732 10.1016/j.prosdent.2013.07.010

[b4] Flügge T. V., Schlager S., Nelson K., Nahles S. & Metzger M. C. Precision of intraoral digital dental impressions with iTero and extraoral digitization with the iTero and a model scanner. Am. J. Orthod. Dentofacial Orthop. 144, 471–478 (2013). 23992820 10.1016/j.ajodo.2013.04.017

[b5] Naidu D. & Freer T. J. Validity, reliability, and reproducibility of the iOC intraoral scanner: A comparison of tooth widths and Bolton ratios. Am. J. Orthod. Dentofacial Orhop. 144, 304–310 (2013). 10.1016/j.ajodo.2013.04.011 23910212

[b6] Cuperus A. M. *et al.* Dental models made with an intraoral scanner: a validation study. Am. J. Orthod. Dentofacial Orthop. 142, 308–313 (2012). 22920696 10.1016/j.ajodo.2012.03.031

[b7] Sousa M. V., Vasconcelos E. C., Janson G., Garib D. & Pinzan A. Accuracy and reproducibility of 3-dimensional digital model measurements. Am. J. Orthod. Dentofacial Orthop. 142, 269–273 (2012). 22858338 10.1016/j.ajodo.2011.12.028

[b8] Mehl A., Ender A., Mörmann W. & Attin T. Accuracy testing of a new intraoral 3D camera. Int. J. Comput. Dent. 12, 11–28 (2009). 19213357

[b9] Persson A., Odén A., Andersson M. & Sandborgh-Englund G. Digitization of simulated clinical dental impressions: Virtual three-dimensional analysis of exactness. Dent. Mater. 25, 929–936 (2009). 19264353 10.1016/j.dental.2009.01.100

[b10] Wang R., Tian W., Wang P. & Wang L. Analysis of vibration effect to surface figure measurement. Acta Optica Sinica 32, 105–108 (2012).

[b11] Jiang H., Zhao H. & Li X. High dynamic range fringe acquisition: A novel 3-D scanning technique for high-reflective surfaces. Opt. Laser Eng. 50 14, 84–1493 (2012).

[b12] Peng S. & Hwang W-L. Adaptive signal decomposition based on local narrow band signals. IEEE Trans. Signal Processing 56 2, 669–2676 (2008).

[b13] Guangshu H. in Digital signal processing, theory, algorithm and realization (ed. 2 Guangshu H. ) Ch. 3, 117–150 (Tsinghua University Press, 2003).

[b14] Gardner F. M. Margins of complete crowns—literature review. J. Prosthet. Dent. 48, 396–400 (1982). 6752383 10.1016/0022-3913(82)90072-5

[b15] Ke H. & Hao Z. in Occlusion: theories & clinical practice (ed. 2 Ke H. & Hao Z. *et al.* ) Ch. 2, 24 (People’s Military Medical Press, 2014).

[b16] Yinghua X. in Practical occlusion (ed. 1 Yinghua X. *et al.* ) Ch. 4, 127. (Scientific and Technical Documents Publishing House, 2011).

